# Use of Chemical Tracers in *Sus scrofa* Population Studies—A Scoping Review

**DOI:** 10.3390/ani14233424

**Published:** 2024-11-27

**Authors:** Grzegorz Tarasiuk, Luis G. Giménez-Lirola, Marisa L. Rotolo, Jeffrey J. Zimmerman

**Affiliations:** 1 College of Veterinary Medicine, Iowa State University, Ames, IA 50011, USA; tarasiuk@iastate.edu (G.T.); luisggl@iastate.edu (L.G.G.-L.); 2 National Pork Board, Des Moines, IA 50325, USA; mrotolo@pork.org

**Keywords:** tracer, marker, *Sus scrofa*, wild boar, feral swine, ethology

## Abstract

A highly invasive species, free-ranging *Sus scrofa* often negatively impact the ecosystem and are capable of spreading a number of impactful pathogens to domestic livestock. Measures taken to ameliorate these impacts and/or control population size are based on the delivery of oral baits containing bioactive chemicals or vaccines, e.g., classical swine fever vaccine. The efficacy of these methods depends on the rate at which inoculated baits are consumed by the pigs. Rhodamine B, tetracycline, and iophenoxic acid are commonly used to quantitate bait uptake in free-ranging pig population studies. All three are effective in this application but differ in fundamental characteristics. When used as a tracer, the effective dose of rhodamine B was established at 15 mg/kg to ensure a 12-week window of detection based on evaluation of hair samples using fluorescent microscopy. Tetracyclines are likewise effective tracers in free-ranging pigs, but the process of detection is highly invasive, i.e., requires euthanasia, and extraction of bone or teeth, followed by examination by fluorescence microscopy. Iophenoxic acid and its derivatives also highly suitable tracers and may be detected in serum for ≥9 months after exposure. Notably tracers used in free-ranging pigs are not suitable for behavioral studies in farm-raised pigs either because the detection method is highly invasive (tetracyclines) or because they are unapproved for use in meat destined for human consumption.

## Simple Summary

Variably known as wild boar, feral pigs, wild hogs, and other names, *Sus scrofa* is a highly invasive species present on every continent except Antarctica. Free-ranging pigs often negatively impact the ecosystem and have the potential to spread impactful pathogens to domestic livestock. Countermeasures taken to control population size and/or reduce the circulation of infectious diseases are based on the delivery of baits containing bioactive chemicals or vaccines, e.g., classical swine fever vaccine. The efficacy of these methods is reliant on the consumption of baits by the pigs and tracers, i.e., rhodamine B, tetracycline, and iophenoxic acid, are commonly used to quantitate bait uptake in free-ranging pig population studies. All three tracers have been shown to be highly effective for this purpose, but, as reviewed herein, their persistence in animals and the methods for detecting their presence in pigs vary. Safer, non-infectious tracers that persist in pigs and can be easily detected through non-invasive methods are needed for population studies in both free-ranging and farm-raised pigs.

## 1. Introduction

The National Cancer Institute (NCI, U.S. National Institutes of Health) defines a tracer as a chemical substance that, by virtue of a unique physical property, can be introduced into a biological or mechanical system and followed through the course of a process [1]. The NCI definition is primarily focused on clinical and molecular applications, but tracers are likewise used in a variety of other studies, including animal behavior and population studies.

Herein, we review orally administered chemical tracers commonly used in studies involving *Sus scrofa* and, more specifically, for evaluating the delivery of oral vaccines in populations of free-ranging swine. Chemical tracers used to study oral bait acceptance by free-ranging pigs are limited to rhodamine B, tetracyclines, and iophenoxic acid [2]. Therefore, a systematic search of the referred literature for the period between 1960 and 2024 was performed on Google Scholar using the following terms: (1) Rhodamine B AND “wild boar” OR “feral pig” OR “wild hog”; (2) Tetracycline AND “wild boar” OR “feral pig” OR “wild hog” WITHOUT antimicrobial resistance; (3) Iophenoxic acid AND “wild boar” OR “feral pig” OR “wild hog” (accessed on 21 August 2024). Review of the titles of the 595 refereed publications produced by this search resulted in 52 relevant publications. Detailed review of these publications and a follow-up snowball search resulted in 16 publications that met the objectives of the review.

## 2. Use of Tracers in Free-Ranging *Sus scrofa*

### 2.1. Free-Ranging Sus scrofa

Variably referred to as wild boar, feral pigs, wild hogs, and other terms [3,4], *Sus scrofa* is a highly invasive species that has successfully adapted to a variety of environments and climates around the world [5,6]. As reviewed by Massei et al. (2004) [7], free-ranging pigs are present on every continent except Antarctica; generally, with negative impacts on the ecosystem, including soil erosion, seed dispersal, and habitat disturbance for ground mammals and birds. In addition, free-ranging pigs, through their rooting behavior damage farmland cause significant harm to cultivated crops [8]. Further, free-ranging pigs have the potential to spread a number of impactful pathogens to domestic livestock, e.g., pseudorabies virus, *Brucella* spp., classical swine fever virus (CSFV), African swine fever virus (ASFV), and foot-and-mouth disease virus (FMDV) [9,10,11,12,13,14]. Introduction of these diseases into farm-raised pigs results in losses in productivity (mortality and morbidity), expenditures for treatment and prevention, and loss of access to international markets [15].

Management of populations of free-ranging pigs may be based on either lethal (removal of animals) or nonlethal strategies [16]. Nonlethal methods include fertility control (injectable contraceptives), fencing, repellents, diversionary feeding, and translocation, but may also include vaccination to reduce the risk of disease spread to farm-raised pigs. Successful oral vaccination of wildlife populations requires delivering a vaccine able to induce protective immunity in a consumable bait that will attract and be accepted by the target species without affecting the non-target species.

This approach can be highly effective. For example, rabies virus was eradicated from fox populations in 10 European countries between 1978 and 2014 using oral vaccine delivered in a fishmeal bait [17]. In free-ranging pigs, oral vaccination programs using live-attenuated CSFV vaccines have been implemented widely, e.g., Belgium, Bulgaria, France, Germany, Hungary, Italy, Japan, Latvia, Luxemburg, Romania, and Slovakia [11,18]. Variables known to affect bait acceptance in free-ranging pig populations include season, bait composition, bait presentation, previous experience with bait, and the presence of non-target species that compete for bait [18].

Ultimately, understanding and optimizing vaccine delivery requires estimates of the rate of bait uptake at the individual pig level. In the case of CSFV, various vaccines have been used (C-strain and Thiverval strain), but none inherently provide the ability to identify vaccinated animals [11]. Alternatively, inclusion of a chemical tracer in the bait and subsequent detection of the tracer in pigs provides the means to evaluate bait uptake.

### 2.2. Rhodamine B

Rhodamine B was first synthesized in 1887 and has been used in many industrial applications, including textiles and printing inks [19]. Since the early 1960s, rhodamine B has been used as an environmental tracer, e.g., river water dispersion experiments, karst ground water tracing, and other hydrological applications [20]. Rhodamine B is well-suited for these applications because it is stable in the environment, it can be detected fluorometrically at concentrations as low as 1 part per 10 billion, and its fluorescence is not degraded by natural light [21,22]. Gast (1963) [22] first proposed the use of rhodamine B in animal studies (mice) and showed that it could be detected in the feces of mice after oral administration. Subsequent studies suggested that rhodamine B was relatively safe in biological systems, e.g., the median lethal dose (LD_50_) for mice (oral administration) was estimated at 887 mg/kg (Rochat, 1978) [23] and Smart (1984) [20] concluded that aquatic ecosystems were not harmed by rhodamine B (1 mg/L) after exposure for 48 h.

Rhodamine B has been used as a tracer in a number of wildlife species including coyotes (*Canis latrans*), European badger (*Meles meles*), stoats (*Mustela ermine*), house mice (*Mus musculus*), and mountain beaver (*Aplodontia rufa*) [24]. Following oral consumption, rhodamine B is deposited in keratinous tissues and, thus, can be visualized under UV light as fluorescent bands in hair, whiskers, and claws [21]. Fluorescent bands appear in hair as soon as 24 h after exposure, but the dose affects the strength of the fluorescence [21]. Likewise, multiple exposures to rhodamine B over time result in multiple fluorescent bands in a hair or whisker with each band corresponding to an exposure event. The persistence of fluorescent bands varies among species and by exposure dose, but has been reported as 119 to 196 days for mountain beaver or 175 days for coyotes [25,26]. As reviewed by Fisher (1999) [21], detection in feces is relatively short (hours) after a single administration of rhodamine B or few days after multiple administrations.

Several studies demonstrated the utility of rhodamine B as a tracer for studies in free-ranging pigs. Morris et al. (2005) [27] described the first use of rhodamine B in free-ranging pigs in a study designed to assess the rate of exposure to brodifacoum, a vitamin K antagonist used to control rats (*Rattus rattus*) in Motatau, New Zealand. Specifically, 6 rat carcasses were injected with a solution containing rhodamine B and placed in the environment. After 8 weeks, 11 pigs were captured, and fluorescent bands were observed in whiskers from 3 of 11 animals, thereby indicating consumption of the rats. Beasley et al. (2015) [24] explored the effect of time on the detection of rhodamine B in guard hair and vibrissae. Following oral administration of rhodamine B (30 mg/kg), fluorescence microscopy of guard hair and vibrissae collected at 7 and 14 days post exposure by found no differences between the two samplings, i.e., 100% of guard hair and 98% of vibrissae positive for rhodamine B.

Baruzzi et al. (2017) [2] evaluated the efficacy of rhodamine B as a marker in free-ranging pigs, with a focus on comparing the detection of rhodamine B in hair from different body regions, assessing the effect of hair growth rates on detection, determining the minimum time between exposure and detection, and identifying the minimum number of hairs required to confirm exposure. Under research conditions, rhodamine B was administered via baits at a dose of 17–20 mg/kg body weight and hair samples were analyzed by fluorescence microscopy. The authors recommended collecting at least two shoulder hairs, two whiskers, and mane and flank hairs to confidently assess exposure to rhodamine B. To differentiate between exposure events, a minimum of 2 to 3 weeks between exposures was needed to observe distinct fluorescence bands in the hair. All exposed pigs still had detectable rhodamine B fluorescent bands in their hair at 49 days post exposure.

Webster et al. (2017) [28] provided guidelines for using rhodamine B in bait for free-ranging pigs, with a focus on determining the minimum detectable dose and its persistence over a 12-week period. In pigs orally administered rhodamine B at the rate of 5, 15, or 30 mg/kg, vibrissae collected at the end of 12 weeks were positive in 1 of 5 pigs in the 5 mg/kg group, 5 of 5 in the 15 mg/kg group, and 5 of 5 in the 30 mg/kg group. Thus, the authors concluded that an oral dose of 15 mg/kg of rhodamine B would be the minimum dose for ensuring 12-week persistence in free-ranging pigs.

Snow et al. (2019) [29] evaluated the use of rhodamine B as a marker of bait uptake in a field study of free-ranging pigs in Texas, USA. Initially, 37 free-ranging pigs were captured and fit with GPS collars to track their movements. Thereafter, the pigs were trained for 7 days to consume bait from feeding stations, after which the feeders were stocked with bait containing rhodamine B (20 mg/kg body weight). Trapping was initiated 15 days later. Among a total of 428 free-ranging pigs from which vibrissae were collected, 317 (74.1%) tested positive for rhodamine B. Among the 37 GPS-collared pigs, 33 visited the bait stations, 18 of which were captured and sampled. Of these, 16 (88.9%) tested positive for rhodamine B.

Cumulatively, these studies showed rhodamine B to be a highly effective, non-invasive marker for tracking bait consumption in free-ranging pigs. A particular advantage of using rhodamine B as a tracer include the ease of sample collection (hair) and evaluation (fluorescence microscopy).

### 2.3. Tetracyclines

Tetracyclines are a class of antibiotics produced by *Streptomyces* spp. and first approved for use in humans by the U.S. Food and Drug Administration in 1948 [30]. Tetracyclines provide broad spectrum antimicrobial activity against Gram-positive bacteria, Gram-negative bacteria, and protozoan parasites [31]. Relevant to their use as tracers, tetracyclines bind to calcium in the body and are subsequently sequestered in bone tissue, i.e., deposited in hydroxyapatite within actively mineralizing bone, hyaline cartilage, and developing teeth [32]. Prolonged exposure to tetracyclines results in a visible yellowish discoloration of the teeth [33]. The presence of tetracyclines in bone and teeth can visualized in both whole specimens and tissue sections using ultraviolet light and even a single exposure can produce a visible yellow band in sections of bone and teeth using fluorescence microscopy [34]. The degree of tetracycline uptake within bone, and hence the strength of fluorescence, is influenced by the age of the animal. Myers and Jaffe (1965) [35] demonstrated that young rats (*Rattus norvegicus*) retained more tetracycline in bone compared to mature rats. In rats, tetracyclines can persist in bones for up to 5–7 months following a single intraperitoneal injection [36,37].

Due to their persistence and detectability, tetracyclines have been used as tracers in numerous animal species, e.g., black bear (*Ursus americanus*), polar bear (*Ursus maritimus*), and white-tailed deer (*Odocoileus virginianus*) [38]. Reidy et al. (2008) [39] evaluated the use of tetracycline hydrochloride (THC) as a tracer in wild boar. Nine boars orally consumed 3 to 13 g THC/kg of body weight, after which they were euthanized at 7 days (*n* = 3), one month (*n* = 3), and 2 months (*n* = 3) post-exposure. To detect the deposition of THC in teeth, the mandibula of the boars were removed and boiled for 3 h. The teeth were then removed, sectioned (100–150 µm), and examined for fluorescence. All boars tested positive for tetracycline deposition, but the markings were faint, leading the authors to recommend a higher dosage for clearer results. Thereafter, Reidy et al. (2011) [38] evaluated the use of THC as a tracer in free-ranging pig populations. After free-ranging pigs became accustomed to consuming corn at bait stations, pigs were provided corn mixed with THC (833 mg THC/kg corn). Among the wild boar (*n* = 125) captured and tested, 41 were positive for THC in their teeth. In a similar fashion, Campbell et al. (2011) [40] pre-baited free-ranging pigs with corn (14 days) followed by corn mixed with fishmeal baits each containing 250 mg of THC (7 days). Among 81 captured wild boar, 73 were positive for THC when the teeth were examined.

Thus, THC is a effective marker for tracking bait consumption in free-ranging pigs, but the detection of THC in individual animals requires euthanasia, extraction of teeth or bones, and extensive processing prior to evaluation by fluorescence microscopy.

### 2.4. Iophenoxic Acid

Iophenoxic acid (IA) was introduced in the 1950s for use in human diagnostics and, specifically, to visualize the gall bladder [41]. That is, IA is metabolized in the liver and excreted into bile, thereby allowing the gall bladder to be visualized radiographically [42]. However, its use in human diagnostic medicine was discontinued in 1957 due to concerns related to IA’s lengthy half-life (~2.5 years) and the prolonged elevation of serum iodine levels induced by IA [43,44]. This long period of detection is useful in wildlife studies and IA and its derivatives (methyl-, ethyl-, and propyl-IA) have been used as tracers in a number of wildlife species, including coyote (*Canis latrans*), dog (*Canis familiaris*), red fox (*Vulpes vulpes*), arctic fox (*Alopex lagopus*), skunk (*Mephitis mephitis*), raccoon (*Procyon lotor*), cat (*Felis catus*), ferret (*Mustela furo*), goat (*Capra hircus*), red deer (*Cervus elaphus scoticus*), white-tail deer (*Odocoileus virginianus*), and rabbit (*Oryctolagus cuniculus*), as reviewed by Ballesteros et al. (2013) [45].

In an early study in free-ranging pigs, Fletcher et al. (1990) [46] compared the use of IA to THC by including both in baits. When bait consumption was determined by detecting the characteristic presence of THC in teeth, i.e., a fluorescent yellow line, 38 of 40 wild boar were positive. When determined by measuring iodine levels in serum, 76 of 80 wild boar showed elevated iodine levels indicative of IA exposure. Thus, Fletcher et al. (1990) [46] reported 100% agreement between the 2 methods, i.e., wild boar positive for THC were also positive for IA exposure and wild boar negative for THC were also negative for elevated serum iodine concentrations.

In swine, IA and its derivatives (methyl-, ethyl-, and propyl-IA) are detectable in serum for ≥9 months after exposure [47,48]. Detection in both research and field studies may be determined indirectly by comparing pre- vs. post-exposure iodine concentrations in serum or plasma using spectrophotometry or directly by measuring IA concentrations in serum or plasma using high-performance liquid chromatography or liquid chromatography electrospray ionization mass spectrometry. When based on serum iodine concentrations, the positive threshold may be calculated as 3 standard deviations above the mean pre-exposure serum iodine concentration [46,49,50]. An advantage of direct measurement is that high-performance liquid chromatography is able to differentiate between IA analogs (ethyl- methyl, and propyl-IA) [47,51,52,53]. While most studies have collected serum and/or plasma for testing, Sage et al. (2013) measured and compared ethyl-, methyl-, and propyl-IA detection in thoracic fluid, muscle, liver and serum. Ethyl- and propyl-IA were detected in serum, liver, and muscle for up to 7 months post-exposure, but not in thoracic fluid.

Several dose-response studies have been conducted under experimental conditions. Cowled et al. (2008) [49] administrated 20, 30, or 40 mg of IA via gavage (3 pigs/treatment group) and, in a follow-up study, offered bait with IA capsules (20 mg). Similarly, Ballesteros et al. (2010) [47] and Sage et al. (2013) [52] orally administrated IA analogs at a dosage of 5 and 15 mg/kg body weight, and 40 mg or 200 mg per pig, respectively. Massei et al., 2009 [51] exposed wild boar to ethyl-IA and propyl-IA (5 mg/kg, 10 mg/kg, and 20 mg/kg) mixed with sugar syrup and placed in baits (4 pigs per treatment group). These studies showed that consumption of IA elevated serum iodine levels by day 3, with a slight decline by days 6 and 8 post exposure [49]. Direct detection of IA analogs showed that ethyl- and propyl-IA were detectable for up to 18 months post-exposure (15 mg/kg), while methyl-IA was detected as long as 9 months post exposure [47]. Similarly, Sage et al., 2013 [52] reported that ethyl- and propyl-IA were detected in serum, liver, and muscle until day 217 post-exposure.

Field studies in which IA and/or IA analogs were placed in gelatin capsules or injected into baits showed it to be an effective method for tracing bait consumption by free-ranging pigs (20 mg to 40 mg) [46,48,49,50,53]. Mitchel et al. (1998) [50] and Cowled et al. (2008) [49] reported elevated iodine levels in 40 of 63 and 15 of 19 pigs, respectively. Ballesteros et al. (2011) [53] reported direct detection of ethyl- and methyl-IA in 11.5% to 56.4% of samples, depending on the population monitored.

Thus, IA is an effective tracer for studies in free-ranging pigs. It is safe, e.g., the median lethal dose (LD_50_) ethyl-IA in orally exposed mice was estimated as 1850 mg/kg body weight and exhibits long persistence [45]. Indeed, offspring of females previously exposed to IA tested positive for IA in serum, indicating transplacental transfer of IA [47].

## 3. Discussion

For field studies that involve voluntary oral consumption of a bait containing a biologic or bioactive chemical, tracers and follow-up testing to identify participant animals are invaluable in assessing the effectiveness of the delivery system. Ideally, chemical tracers used in such studies will have some or all of the following characteristics [2]: (1) low cost, (2) stability throughout the process of exposure, digestion, and detection, (3) acceptance (palatability) by the target species, (4) no adverse ecological effects, (5) no adverse health effects in target or non-target species, (6) detectable using standard laboratory procedures, (7) testing data is informative regarding the time interval since the tracer was ingested, and (8) a quantitative testing result. In free-ranging pigs, tracers that meet some or most of these characteristics are limited to rhodamine B, tetracyclines, and iophenoxic acid.

Rhodamine B is a low-cost and widely available dye which is highly stable over a range of environmental conditions [54]. In field applications, rhodamine B has a neutral palatability and does not affect bait consumption by pigs, either positively or negatively [55]. Sample collection (hair) is simple and non-invasive, i.e., can be done on a conscious animal with minimal restraint. Testing is done on clean hair samples with positive samples showing fluorescent bands. Quantitation and time since exposure is inexact, but separate exposure events can be identified as separate fluorescent bands, if the exposures were ≥2 weeks apart. However, it should be borne in mind that hair growth patterns may affect the fluorescence response [2]. Although rhodamine B is not a genotoxic and/or mutagenic hazard, the impurities in the commercial product may be toxic [56]; for which reason the safety of rhodamine B remains a point of discussion. The use of rhodamine B in foods has been prohibited in many countries and its application in free-ranging population studies is a food safety consideration for people who consume wild boar [57].

Tetracyclines are low cost and widely accessible antibiotics with a shelf life estimated at 6.2 to 29.7 months when stored at room temperature [58]. Tetracyclines are compatible with oral baits and readily consumed by free-ranging pigs [39]. Notably, absorption of tetracyclines in the gastrointestinal tract is affected by the presence of proteins, carbohydrates, and fat, hence 25% and 60% of the product consumed is bioavailable [59,60]. However, sampling for detecting the presence of tetracycline is highly invasive, i.e., requires euthanasia, collection of bone and/or teeth for sectioning, and evaluation using fluorescence microscopy. Separate exposures to tetracycline may be visualized as distinct fluorescent bands, but there is little data on interpretation of temporal effects [61]. Tetracyclines are safe for free-ranging pigs and may be administered both orally and parenterally. However, their use as tracers in free-ranging pigs could present a risk to humans who unknowingly consume meat containing tetracycline residues [62].

Similarly, IA is a low-cost chemical which is highly stable over a range of environmental conditions [52]. IA is palatable for pigs, i.e., inclusion of IA in baits did not affect consumption [45], but the size of the capsules used to incorporate IA into baits did affect uptake [49]. Detection of IA is invasive, i.e., requires blood collection, and requires anesthesia or euthanasia of animals. Testing for exposure to IA may be done indirectly by measuring iodine concentration or directly by detecting IA or IA analogs in serum [45]. In either case, testing for IA is quantifiable, i.e., the higher the exposure dose, the higher concentration of IA in serum and the longer its persistence [52]. IA is non-toxic agent to wildlife [42] and humans, but consumption of pork containing IA could result in persistently elevated iodine levels in humans [52].

## 4. Conclusions

Rhodamine B, tetracyclines, and iophenoxic acid have been shown to be valuable in studying populations of free-ranging *Sus scrofa* and their acceptance of orally-delivered vaccines or bioactive chemicals. However, with the exception of tetracyclines for which withdrawal periods have been established, rhodamine B and iophenoxic acid are not approved for use in animals intended for human consumption. By extension, rhodamine B and iophenoxic acid are not suitable for use in population studies involving farm-raised pigs intended to enter the food chain. Tetracyclines, on the other hand, could be used under specific circumstances, but the requirement for visualizing fluorescent bands in teeth or bone is impractical and limits its application. Therefore, there is a need for safer, non-infectious tracers that persist in animals and are easily detected using non-invasive methods for use in population studies involving free-ranging and farm-raised pigs.

## Data Availability

No additional data is available.
